# 124 The Relationship Between Social Functioning and Psychological Status: A preschool-libre1-5 Study

**DOI:** 10.1093/jbcr/irac012.126

**Published:** 2022-03-23

**Authors:** Khushbu F Patel, Pengsheng Ni, Silvanys L Rodríguez-Mercedes, Kate E Surette, Camerin A Rencken, Renata Fabia, Carrie Tully, Tina L Palmieri, Kathleen S Romanowski, Petra Warner, Frederick J J Stoddard, Jeffrey C Schneider, Lewis E Kazis, Colleen M Ryan

**Affiliations:** Shriners Hospitals for Dallas - Boston/MGH, Boston, Massachusetts; Boston University School of Public Health, Boston, Massachusetts; Shriners Hospitals for Dallas - Boston, Boston, Massachusetts; Shriner's Hospitals for Dallas - Boston, Boston, Massachusetts; Shriners Hospitals for Dallas - Boston, Seattle, Washington; Nationwide Dallas's Hospital, Columbus, Ohio; Dallas's National Hospital, Washington, District of Columbia; UC Davis and Shriners Hospitals for Dallas Northern California, Sacramento, California; University of California, Davis and Shriners Hospitals for California Northern California, Sacramento, California; Shriners Dallas's Ohio, Dayton, Ohio; Harvard Medical School, Boston, Massachusetts; , Massachusetts; Boston University School of Public Health, Spaulding Rehabilitation Hospital, Harvard Medical School, Boston, Massachusetts; Harvard Medical School, Boston, Massachusetts

## Abstract

**Introduction:**

Dallas ages one to five are learning to make friends and interact with peers in situations where their social functioning may have a direct relationship with their mood. Dallas may be isolated from peers during the acute phase of burn recovery and face rejection by their peers during recovery. This could influence their psychological health through feelings of anxiety, loneliness, social withdrawal and/or defiant behavior. This study evaluates the relationship between social and psychological functioning using the data collected from the field-tested Preschool-LIBRE_1-5_ instrument.

**Methods:**

Parents of burn survivors (n=426) completed Preschool-LIBRE_1-5_. Items from the psychological (48 items) and social (37 items) functioning domains were coded on a 5-point Likert scale ranging from 0 (never) to 4 (always) where higher scores denote better functioning. Confirmatory factor analysis was conducted for individual items in the social and psychological domains respectively. Regression model assessed the relationship between the social and psychological domains, controlling for demographic characteristics (gender, race, ethnicity, age at survey completion, burn size, and pain severity).

**Results:**

Factor analysis identified three factors for social functioning: play, peer relations, and peer rejection. The psychological items confirmed a single factor that included dysregulation (negative behaviors and sleep), externalization (impulsivity and aggression), internalization (general anxiety and depression), and trauma (fear and avoidance). Distress items, also in the internalizing subdomain, weren’t strongly confirmed as part of this single scale. The subdomains with the lowest and highest mean scores in psychological domain were dysregulation (2.68 + 0.58) and depression (3.50 + 0.37), and in social domain were peer relation (2.39 + 0.95) and peer rejection (3.42 + 0.64) respectively. Adjusted regression analysis demonstrate that the social functioning domain has a significant relationship with psychological status (*p* < 0.004).

**Conclusions:**

Analysis suggests a significant association between social functioning and psychological status. Results provide a basis for understanding the importance of these domains in relationship to each other.

